# Identification of a threshold to discriminate fasting hypertriglyceridemia with postprandial values

**DOI:** 10.1186/s12944-018-0803-8

**Published:** 2018-07-18

**Authors:** Magdalena del Rocío Sevilla-González, Carlos A. Aguilar-Salinas, Liliana Muñóz-Hernández, Paloma Almeda-Valdés, Roopa Mehta, Rafael Zubirán, Omar Yaxmehen Bello-Chavolla, Donaji V. Gómez-Velasco, Arsenio Vargas-Vázquez, Tannia Viveros-Ruíz, Alexandro J. Martagón-Rosado, Ivette Cruz-Bautista

**Affiliations:** 1 Unidad de Investigación de Enfermedades Metabólicas, Instituto Nacional de Ciencias Médicas y Nutrición Salvador Zubirán, Vasco de quiroga 15, 14200 México, México; 2 Programa de Doctorado en Ciencias Médicas y de la Salud, Universidad Nacional Autonóma de México, México, México; 3 Departamento de Endocrinología y Metabolismo, Instituto Nacional de Ciencias Médicas y Nutrición Salvador Zubirán, México, Mexico; 4 Tecnológico de Monterrey, Escuela de Medicina y Ciencias de la Salud, Monterrey, N.L Mexico; 5 MD/PhD (PECEM) Program, Facultad de Medicina, Universidad Nacional Autónoma de México, México, México; 6 Cátedra Conacyt, México, México

**Keywords:** Postprandial triglycerides, Hypertriglyceridemia, Cardiovascular risk

## Abstract

**Background:**

Postprandial lipemia is an important cardiovascular risk factor. The assessment of postprandial lipid metabolism is a newly trend that several consortiums and countries have adopted. The aim of the study is to determine a postprandial triglyceride concentration cut-off point that accurately discriminate individuals with fasting normal triglyceride concentrations from those with fasting hypertriglyceridemia.

**Methods:**

Cross sectional population-based study. A total of 212 subjects underwent an eight hours’ oral fat tolerance test. Samples were taken fasting, three, four, five, six and eight hours after the meal. The area under the receiver operating characteristic curve (*c-statistic*) was computed using postprandial triglycerides concentrations as independent predictor, and fasting hypertriglyceridemia as dependent variable.

**Results:**

The best threshold of postprandial lipemia to discriminate fasting hypertriglyceridemia was 280 mg/dL at any hour area under the curve 0.816 (95% confidence interval 0.753–0.866), *bootstrap-corrected c-statistic* = 0.733 (95% confidence interval 0.68–0.86). The same value was compared with apolipoprotein B concentrations (>90th percentile) having a good performance: area under the curve 0.687 95% confidence interval 0.624–0.751). Likewise, subjects with high postprandial lipemia have higher Globo risk scores.

**Conclusion:**

The 280 mg/dL cut-off point value of postprandial triglycerides concentration any time after a test meal discriminate subjects with fasting hypertriglyceridemia. This threshold has a good performance in a heterogeneous population and has a good concordance with cardiovascular risk surrogates.

## Background

Postprandial lipemia has been established as an important cardiovascular risk factor [1–3]. Remnants production, inflammation process, and the resulting endothelial dysfunction are the primary mechanisms which trigger cardiovascular risk in the postprandial state [4, 5]. Metabolic conditions (type 2 diabetes mellitus, obesity/overweight [6]), age, gender and genetic background [4, 7, 8] determine postprandial triglyceride (TG) response.

The assessment of the postprandial plasma lipid concentrations has recently been recommended by several consortiums [9–11] as an approach to evaluate the lipid profile. The measurement of TG in the postprandial state represents several advantages over fasting levels. Subjects are in a postprandial state for most of the day, which allows blood sampling without the need for fasting evaluations. Since there is biological variation in the postprandial TG response, it is imperative to establish a population specific cut-off point. The identification of such threshold would be clinically convenient, since it would allow the diagnosis of fasting abnormal concentration utilizing a postprandial sample. In this study, we aim to show that a postprandial TG cut-off point can accurately discriminate individuals with normal fasting triglyceride levels from those with fasting hypertriglyceridemia.

## Methods

### Study participants

We performed a cross-sectional evaluation in volunteers, who attended at the Endocrinology Department of the institution after a phone call invitation. Inclusion criteria included Mexican adults aged between 18 and 77 years old with or without lipid disorders, but without use of lipid lowering drugs for at least 6 weeks prior to entering the study. Exclusion criteria included a TG concentration > 1000 mg/dL following a 12 h fast, acute or chronic diseases that could modify lipid concentrations, pregnancy, cigarette consumption (defined as > 15 cigarettes per day), alcohol consumption (> 2 servings per day/7 days a week), and strenuous exercise (more than three times per week). Written informed consent was obtained from each participant. Investigation was conducted according to the principles expressed in the Helsinki Declaration of Human Studies.

### Oral fat tolerance test

After a 12 h fast, all subjects underwent an oral fat tolerance test (OFTT). A standardized high fat meal was given to each subject. This consisted of a (Mc Donald’s) quarter pounder hamburger with cheese, whole milk (240 ml), 5 g of mayonnaise, and Mc Donald’s French fries (70 g). The meal contained 52 g of fat, (15 g of saturated fat, 14.68 g monounsaturated fat, 9.96 g polyunsaturated fat, 104.19 mg cholesterol) 75 g of carbohydrates, 40 g of protein, equal to 960 kcal. Blood samples were drawn before and 3, 4, 6 and 8 h after the meal. Subjects remained seated throughout the study.

### Biochemical and sample analysis

Blood samples were drawn from the dominant arm and collected in EDTA- containing tubes (BD vacutainer TM, London, UK). The serum was separated by centrifugation for 15 min at 3000 rpm at 4 °C. Serum concentration of TG, total cholesterol, high-density lipoprotein cholesterol (HDL-c) and glucose, were measured by automated enzymatic assays (Beckman Synchron CX, Brea, CA, USA). Low-density lipoprotein cholesterol (LDL-c) was calculated with the Friedewald [12] formula when fasting TG were < 300 mg/dL. Apo-B levels were determined by kinetic nephelometry (Beckman Immage, Brea, CA and insulin concentrations were analyzed utilizing micro particles enzyme immunoassay (MEIA) (Abbot AxSYM System, Green Oaks, IL, USA).

### Statistical analysis

Data were analyzed using R Studio software version 3.4.3. Quantitative variables were examined for normality with the Kolmogorov-Smirnov test and reported as means and standard deviation (±SD) or median and interquartile range (IQR) as appropriate. Categorical variables were reported as frequencies and percentages.

The optimal threshold for postprandial triglycerides was determined utilizing the *Optimal Cutpoints* package [13] computing the Youden Index (sum of sensitivity plus specificity minus 1). Hourly postprandial triglycerides concentrations (3, 4 and 6 h) were evaluated in each hour as independent predictors, and fasting hypertriglyceridemia was the dependent variable. The values were rounded to 5 or 10 mg/dL, their performance was tested at any time of the curve using the *pROC* package [14] to compute the area under the receiver operating characteristic (ROC) curve (*c-statistic*) selecting the point with optimal balance of sensitivity and specificity.

To correct overfitting and quantify optimism sensitivity and specificity of the thresholds given were computed with 2000 stratified bootstrap replicates with a 95% confidence intervals (CI).

## Results

**Fig. 1:**
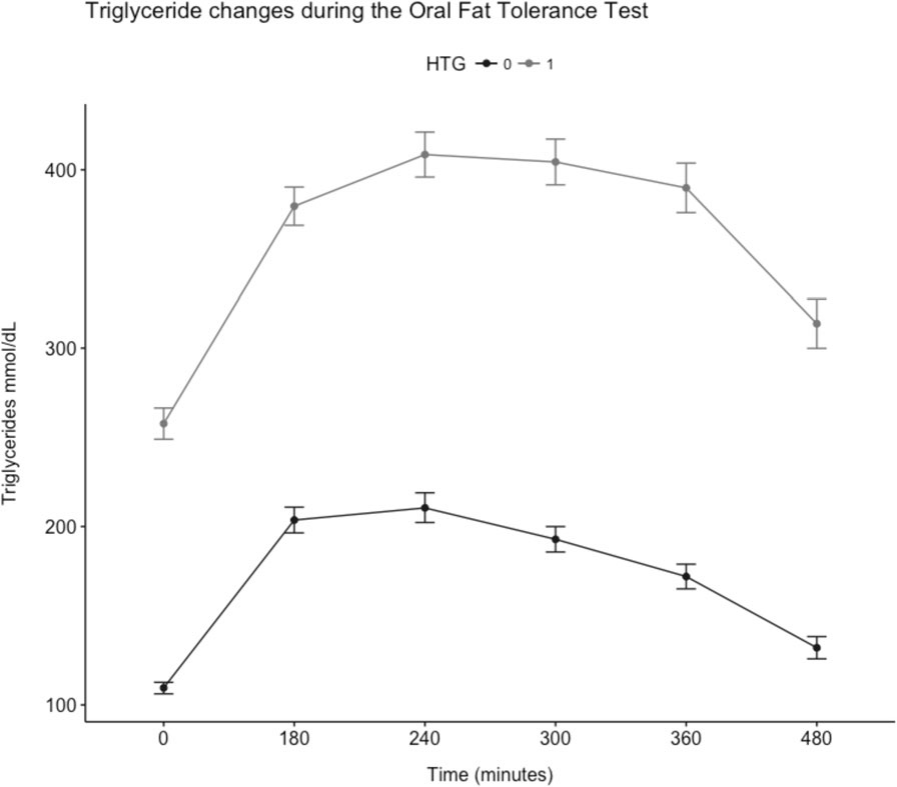
Triglyceride changes during the Oral Fat Tolerance Test in subjects with or without hypertriglyceridemia. Triglyceride changes during the Oral Fat Tolerance Test stratified by fasting hypertriglyceridemia. 0 Absent, 1 Present

A total of 212 subjects underwent the OFTT, 143 (67.5%) women (Fig. 1). Age ranged between 20 and 77 years. Most of the subjects were obese or overweight (74.2%). The dyslipidemia etiologies included primary forms of hypertriglyceridemia (fasting TG > 150 mg/dL) (64%), familiar combined hyperlipidemia (1) LDL-c > 160 mg/dL and /or TG > 150 mg/dL + Apo-B concentrations >90th percentile for Mexican population + first-degree relative with hyperlipidemia) (34.9%) and hypoalphalipoproteinemia (HDL-c < 40 mg/dL ruling out secondary causes) (32.6%). Only two subjects had fasting TG levels above 600 mg/dL. Also, 30 subjects with T2D were included. Clinical and biochemical characteristics of the study population are shown in Table 1.

**Table 1:** Baseline characteristics (*n* = 212) of the studied population

Age (years)	47.51 ± 12.3
Women	143 (67.5%)
BMIª	27.9 ± 4.1
Waist (cm)	92.1 ± 11.0
Waist-hip ratio	0.90 ± 0.07
Fasting glucose (mg/dL)	101 (93–111)
Fasting insulin (mU/L)	8.70 (6.3–12.2)
Total cholesterol (mg/dL)	208.9 ± 38.4
Fasting triglycerides (mg/dL)	183 (130–246)
HDL-c (mg/dL)	44.8 ± 11.8
Aapo-B (mg/dL)	106.6 ± 25.1
LDL-c (mg/dL)	127.6 ± 33.1

^a^*BMI* (Body Mass Index), *HDL-c* (high density lipoprotein cholesterol), *LDL-c* (low density lipoprotein cholesterol)

**Fig. 2:**
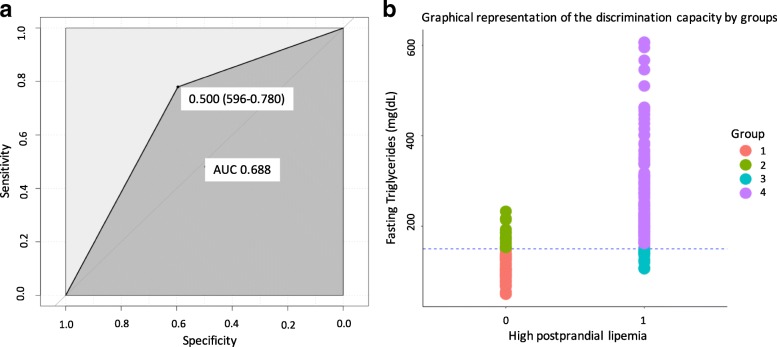
Performance of the postprandial triglycerides value (280 mg/dL) of to discriminate fasting hypertriglyceridemia. **a** Area Under the ROC curve 0.810 (IC_95%_ 0.773–0.847) bootstrap-corrected c-statistic = 0.733 (IC_95%_ 0.68–0.86) (**b**) Representation of the discrimination capacity by groups: 1.- True negative: Normo-triglyceridemia (< 150 mg/dL) /Normo postprandial lipemia (< 280 mg/dL), 2.- False negative: Hypertriglyceridemia (> 150 mg/dL) /Normo postprandial lipemia (< 280 mg/dL) 3.- False positive: Normo-triglyceridemia (< 150 mg/dL) / High postprandial lipemia (> 280 mg/dL) 4.-True positive: Hypertriglyceridemia (> 150 mg/dL) / High postprandial lipemia (> 280 mg/dL). *(Two column fitting image)*

The most suitable threshold of postprandial lipemia to discriminate fasting hypertriglyceridemia was 272 mg/dL at six-hour after the fat load (AUC 0.845, 95% confidence interval CI 0.800–0.80) with a sensitivity (Sens) of 74.4%, and specificity (Spec) 94.6%, positive predictive value (PPV) 0.94 and negative predictive value (NPV) 0.66, Spearman correlation = 0.88 *p = 0.001*. The value was rounded up for clinical practicality to 280 mg/dL. Its performance was tested at any point along the curve. It showed a good performance with an AUC 0.816 (95% CI 0.753–0.866), bootstrap-corrected c-statistic = 0.733 (95% CI 0.68–0.86). The Sens was 84.6%, the Spec 77.3%, the PPV 0.872, the NPV 0.734, the positive likelihood ratio (LR+) 3.74, the negative likelihood ratio (LR-) 0.197, accuracy (ACC) 0.820 and the diagnostic odds ratio 18.98 (Fig. 2). We stratified the study population by weight- and age-groups or gender to evaluate the performance of the new cut-off in specific states. The accuracy remained adequate in all population subgroups (Table 2).

**Table 2:** Accuracy of postprandial lipemia cut-off points to discriminate fasting hypertriglyceridemia in overall and stratified populations

Cut-off point	Population	N	AUC	95% confidence interval	Sen	Spec	PPV	NPV
280 mg/dL	Normal	52	0.831	(0.725–0.936)	0.850	0.812	0.739	0.896
	Overweight-Obesity	147	0.778	(0.699–0.857)	0.831	0.725	0.890	0.617
	≤ 49 years	106	0.816	(0.739–0.894)	0.833	0.800	0.873	0.744
	> 50 years	98	0.786	(0.697–0.875)	0.846	0.727	0.859	0.705
	Women	143	0.787	(0.715–0.858)	0.824	0.750	0.852	0.709
	Men	69	0.858	(0.767–0.95)	0.891	0.826	0.911	0.791
200 mg/dL [15]	Overall	212	0.700	(0.644–0.755)	0.90	0.400	0.752	0.900
180 mg/dL [16]		212	0.593	(0.548–0.637)	0.90	0.186	0.691	0.900
175 mg/dL [17]		212	0.593	(0.548–0.637)	0.90	0.186	0.691	0.900

^a^*AUC* area under the ROC curve, *Sen* sensibility, *Spec* specificity, *PPV* positive predictive value, *NPV* negative predictive value

The performance of other thresholds previously described in the literature [15–17] was also tested in our population and it is shown in Table 2. The 280 mg/dL cut-off point has a better performance than the other plasma triglycerides concentrations to detect fasting hypertriglyceridemia.

The accuracy of the postprandial value was replicated in a separate sample of 71 subjects obtained from the same source of subjects than the discovery cohort, whose characteristics are described in Table 3.

**Table 3:** Baseline characteristics of sample replication (*n* = 71)

Age (years)	47.7 ± 13.66
Women	38 (53.5%)
BMI ª	28.5 ± 5
Waist (cm)	93.9 ± 11.5
Waist-hip ratio	0.89 ± 0.13
Fasting glucose (mg/dL)	102 (94–112)
Fasting insulin (mU/L)	9.0 (5.7–13.9)
Total cholesterol (mg/dL)	215.2 ± 46.0
Fasting triglycerides (mg/dL)	178 (127–241)
Hypertriglyceridemia	41 (57.7%)
Familiar combined hyperlipidemia	23 (32.8%)
Hypoalphalipoproteinemia (< 40 mg/dL)	26 (37.6%)
apo B (mg/dL)	113.7 ± 31.0
HDL-c (mg/dL)	45.0 ± 13.2
LDL-c (mg/dL)	128.6 ± 34.1

^a^*BMI* body mass index), *HDL-c* (high density lipoprotein cholesterol), *LDL-c* (low density lipoprotein cholesterol). Hypertriglyceridemia (> 150 mg/dL)

The AUC was 0.892 (95%CI 0.816–0.967) bootstrap-corrected c-statistic = 0.833 95% CI 0.7–0.96), Sens 95.1%, Spec 83.3%, PPV 0.886, NPV 0.925.

**Fig. 3:**
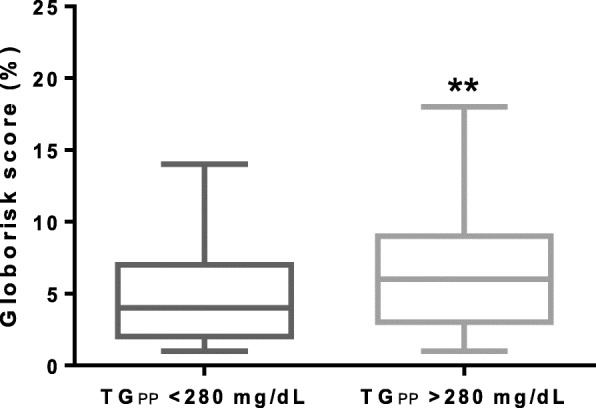
Globorisk scores from patients with high postprandial lipemia. Median Globorisk scores from patients with high postprandial lipemia (> 280 mg/dL) compared to patients with normo postprandial lipemia (< 280 mg/dL) (*p* < 0.01)

In a secondary analysis, we explore the concordance of the new cut-off point with cardiovascular-risk markers. We determine the postprandial triglyceride value to discriminate subjects with abnormal fasting ApoB concentrations (>90th percentile in Mexican adults, 100 mg/dL [18]). The most suitable cut-off point was 261 mg/dL at four-hour (AUC 0.707, Sens 76%, Spec 61.7%, PPV 0.734 and NPV 0.696). At any point along the curve the best cut-off concentration was 280 mg/dL, which correlates with the cut-off point to discriminate fasting hypertriglyceridemia: AUC 0.687 (95% CI 0.624–0.751) Sens 77.9%, Spec 59%, PPV 0.718 and NPV 0.670. We then compared whether our identified cut-off point identified patients at higher cardiovascular risk evaluated by Globorisk score [19]. As shown in Fig. 3, patients with postprandial TG levels > 280 mg/dL had higher median Globorisk scores compared to patients with postprandial TG levels < 280 mg/dL at all time points (*p* < 0.01).

## Discussion

The evaluation of the fasting lipid profile reduces the variability inherent to the use of the Friedewald formula. However, over the past two decades, several consensus panels have recognized the utility of non-fasting samples in the assessment of lipid profiles [9–11] for the evaluation of a patient with dyslipidemia. This study proposes a cut-off point for postprandial triglycerides (280 mg/dL) that identifies subjects with fasting hypertriglyceridemia. Here, we analyzed the results of oral fat tolerance tests (50 g fat challenge), in subjects submitted with a wide range of fasting TG (50-607 mg/dL) levels, in order to establish cut-off points in a controlled sitting, analyzing the values at different time points of the test. Our results demonstrate that a triglyceride concentration of 280 mg/dL at any time point between 3 and 8 h after a meal has a good diagnostic performance in terms of sensitivity (84.6%), specificity (77.3%), and an AUC 0.820 to discriminate subjects with fasting hypertriglyceridemia (> 150 mg/dL) and serves a good estimator of abnormal apo-B concentrations, which makes it a useful marker of cardiovascular disease [20].

Panels of experts have proposed cut-off points of ≥ 200 mg/dL [15], and ≥ 180 mg/dL [16] as optimal thresholds for non-fasting hypertriglyceridemia. The basis under the determination processes of these cut-off points are unclear. The only cut-off point validated in a prospective cohort with cardiovascular outcomes is that stablished by White [17] in the Women’s Health study (175 mg/dL). These thresholds demonstrated a good sensitivity and poor specificity in our population. The performance of the cut-off point of the Athens panel [16] was not different from the Women’s Health (WHI) study. On their behalf, American Heath Association’s cut-off point proved a slightly better performance in our population. These differences in the performance could be given by the quantity of fat intake, gender, ethnicity, and outcome studied. The study sample of the WHI study was composed by women, mainly Caucasians and the primary outcome was cardiovascular events, with no standardization of the meal consumption. The average of postprandial triglyceride increase after a meal with low fat content (15 g) was 20% [21] above the fasting levels, whereas moderate to high fat consumption (50 g) same as we considerate as regular intake in our population, is associated with a 50% increase over the fasting concentrations [22].

Evidence from epidemiological, mechanistic and clinical studies support a causal relation of lifelong exposure to triglyceride rich lipoproteins (TRL’s) and their remnants with the onset of cardiovascular disease (CVD) independent of the HDL-c concentrations [6]. In 1979 the atherogenic properties of the postprandial state were described for first time [23]. Atherogenic burden associated with postprandial lipemia is due to the very low density lipoproteins (VLDL) overproduction or decrease catabolism of TRL’s. During the postprandial state TRL’s penetrate the arterial wall causing foam cell formation [24], triggering an overproduction of inflammatory markers (TNF-alpha [25, 26], interleukin-6 [25, 26], vascular cell adhesion molecule-1 (VCAM-1) [25], and high-sensitivity C-reactive protein (hsCRP) [26]) and oxidative stress [6]. This endothelial dysfunction is an initial process of atherogenesis and contributes to the pathogenesis of CVD [27].

Postprandial triglycerides measurement represents several advantages compared with fasting evaluations. People are in a postprandial state during the major part of the day. The non-requirement for overnight fasting decreases the number of patients during the early hours in laboratories. The threshold reported here will help physicians to identify cases with fasting hypertriglyceridemia, without the need of a fasting sample and a second visit.

The strengths of the study lie in the selection of the participants: wide range of metabolic conditions: dyslipidemias, glycemic and body mass index status, allows to test whether the cut-off point is accurate in a real and diverse scenario. Besides, the controlled homogeneous times and quantity of fat (enough to trigger the stimulus), allows to determine accurately the response. The limitation of the study is that we cannot test the performance of the new value with a lower fat content and most important that the new cut-off point cannot be associated with cardiovascular events or mortality outcomes. These results open the gate for new longitudinal study that prospectively can prove the predictive capacity in mortality and cardiovascular outcomes of the new value.

## Abbreviations

ACC: Accuracy
Apo-B: Apolipoprotein B
AUC: Area under the curve
CI: Confidence intervals
CVD: Cardiovascular disease
HDL-c: High density lipoprotein cholesterol
hsCRP: High-sensitivity C-reactive protein
IQR: Interquartile range
LDL-c: Low density lipoprotein cholesterol
NPV: Negative predictive value
OFTT: Oral fat tolerance test
PPV: Positive predictive value
ROC: Receiver operating characteristic
SD: Standard deviation
TG: Triglycerides
TRL’s: Triglyceride rich lipoproteins
VCAM-1: Vascular cell adhesion molecule-1
